# Clinical Features and Natural History of Preadolescent Nonsyndromic Hypertrophic Cardiomyopathy

**DOI:** 10.1016/j.jacc.2022.03.347

**Published:** 2022-05-24

**Authors:** Gabrielle Norrish, Aoife Cleary, Ella Field, Elena Cervi, Olga Boleti, Lidia Ziółkowska, Iacopo Olivotto, Diala Khraiche, Giuseppe Limongelli, Aris Anastasakis, Robert Weintraub, Elena Biagini, Luca Ragni, Terence Prendiville, Sophie Duignan, Karen McLeod, Maria Ilina, Adrian Fernandez, Chiara Marrone, Regina Bökenkamp, Anwar Baban, Peter Kubus, Piers E.F. Daubeney, Georgia Sarquella-Brugada, Sergi Cesar, Sabine Klaassen, Tiina H. Ojala, Vinay Bhole, Constancio Medrano, Orhan Uzun, Elspeth Brown, Ferran Gran, Gianfranco Sinagra, Francisco J. Castro, Graham Stuart, Hirokuni Yamazawa, Roberto Barriales-Villa, Luis Garcia-Guereta, Satish Adwani, Katie Linter, Tara Bharucha, Esther Gonzales-Lopez, Ana Siles, Torsten B. Rasmussen, Margherita Calcagnino, Caroline B. Jones, Hans De Wilde, Toru Kubo, Tiziana Felice, Anca Popoiu, Jens Mogensen, Sujeev Mathur, Fernando Centeno, Zdenka Reinhardt, Sylvie Schouvey, Perry M. Elliott, Juan Pablo Kaski

**Affiliations:** a Centre for Inherited Cardiovascular Diseases, Great Ormond Street Hospital, London, United Kingdom; b Institute of Cardiovascular Sciences, University College London, London, United Kingdom; c The Children’s Memorial Health Institute, Warsaw, Poland; d Careggi University Hospital, Florence, Italy; e Necker–Enfants Malades Hospital, Paris, France; f Inherited and Rare Cardiovascular Disease Unit, AO dei Colli Monaldi Hospital, Universita della Campania “Luigi Vanvitelli,” Naples, Italy; g Onassis Cardiac surgery Center, Athens, Greece; h The Royal Children’s Hospital, Melbourne, Victoria, Australia; i Cardiology Unit, St Orsola Hospital, IRCCS Azienda Ospedalierao–Universitaria di Bologna, Bologna, Italy; j Our Lady’s Children’s Hospital, Dublin, Ireland; k Royal Hospital for Children, Glasgow, United Kingdom; l Fundación Favaloro University Hospital, Buenos Aires, Argentina; m Papa Giovanni XXIII Hospital, Bergamo, Italy; n Fondazione Toscana G. Monasterio, Massa-Pisa, Italy; o Leiden University Medical Center, Leiden, the Netherlands; p Bambino Gesu Hospital, Rome, Italy; q University Hospital Motol, Prague, Czech Republic; r Royal Brompton and Harefield NHS Trust, London, United Kingdom; s Sant Joan de Deu, Barcelona, Spain; t Department of Pediatric Cardiology, Charite–Universitatsmedizin Berlin, Berlin, Germany; u Experimental and Clinical Research Center, a joint cooperation between the Charité Medical Faculty and the Max-Delbrück-Center for Molecular Medicine, Charite–Universitatsmedizin Berlin, Berlin, Germany; v DZHK (German Center for Cardiovascular Research), partner site Berlin, Berlin, Germany; w Department of Pediatric Cardiology, Pediatric Research Center, New Children’s Hospital, University of Helsinki and Helsinki University Hospital, Helsinki, Finland; x Birmingham Children’s Hospital, Birmingham, United Kingdom; y Hospital General Universitario Gregorio Marañón, Madrid, Spain; z University Hospital of Wales, Cardiff, United Kingdom; aa Leeds General Infirmary, Leeds, United Kingdom; bb Vall d’Hebron University Hospital, Barcelona, Spain; cc Cardiothoracovascular Department, University of Trieste, Trieste, Italy; dd University Hospital Virgen de la Arrixaca, Murcia, Spain; ee Bristol Royal Hospital for Children, Bristol, United Kingdom; ff Department of Pediatrics, Faculty of Medicine and Graduate School of Medicine, Hokkaido University Hospital, Sapporo, Japan; gg Complexo Hospitalario Universitario A Coruña, INIBIC, CIBERCV, La Coruña, Spain; hh University Hospital La Paz, Madrid, Spain; ii John Radcliffe Hospital, Oxford, United Kingdom; jj Glenfield Hospital, Leicester, United Kingdom; kk Southampton General Hospital, Southampton, United Kingdom; ll Hospital Universitario Puerta de Hierro Majadahonda, Madrid, Spain; mm Aarhus University Hospital, Aarhus, Denmark; nn Fondazione IRCCS Ca Granda – Ospedale Maggiore Policlinico Milano, Department di Medicina Interna – UOC Cardiologica, Milan, Italy; oo Alder Hey Children’s Hospital, Liverpool, United Kingdom; pp Ghent University Hospital, Ghent, Belgium; qq Kochi Medical School Hospital, Kochi, Japan; rr Mater Dei Hospital, Msida, Malta; ss University of Medicine and Pharmacy “Victor Babes” Timisoara, Department of Pediatrics, Children’s Hospital “Louis Turcanu,” Timisoara, Romania; tt Aalborg University Hospital, Aalborg, Denmark; uu Evelina Children’s Hospital, London, United Kingdom; vv Rio Hortega University Hospital, Valladolid, Spain; ww The Freeman Hospital, Newcastle, United Kingdom; xx Hospital Saint Joseph, Marseille, France; yy St Bartholomew’s Centre for Inherited Cardiovascular Diseases, St Bartholomew’s Hospital, West Smithfield, London, United Kingdom

**Keywords:** age, childhood hypertrophic cardiomyopathy, outcomes, phenotype, HCM, hypertrophic cardiomyopathy, ICD, implantable cardioverter-defibrillator, IPHCC, International Paediatric Hypertrophic Cardiomyopathy Consortium, LP, likely pathogenic, LV, left ventricular, LVOT, left ventricular outflow tract, LVOTO, left ventricular outflow tract obstruction, P, pathogenic, SCD, sudden cardiac death, VT, ventricular tachycardia

## Abstract

**Background:**

Up to one-half of childhood sarcomeric hypertrophic cardiomyopathy (HCM) presents before the age of 12 years, but this patient group has not been systematically characterized.

**Objectives:**

The aim of this study was to describe the clinical presentation and natural history of patients presenting with nonsyndromic HCM before the age of 12 years.

**Methods:**

Data from the International Paediatric Hypertrophic Cardiomyopathy Consortium on 639 children diagnosed with HCM younger than 12 years were collected and compared with those from 568 children diagnosed between 12 and 16 years.

**Results:**

At baseline, 339 patients (53.6%) had family histories of HCM, 132 (20.9%) had heart failure symptoms, and 250 (39.2%) were prescribed cardiac medications. The median maximal left ventricular wall thickness *z*-score was 8.7 (IQR: 5.3-14.4), and 145 patients (27.2%) had left ventricular outflow tract obstruction. Over a median follow-up period of 5.6 years (IQR: 2.3-10.0 years), 42 patients (6.6%) died, 21 (3.3%) underwent cardiac transplantation, and 69 (10.8%) had life-threatening arrhythmic events. Compared with those presenting after 12 years, a higher proportion of younger patients underwent myectomy (10.5% vs 7.2%; *P* = 0.045), but fewer received primary prevention implantable cardioverter-defibrillators (18.9% vs 30.1%; *P* = 0.041). The incidence of mortality or life-threatening arrhythmic events did not differ, but events occurred at a younger age.

**Conclusions:**

Early-onset childhood HCM is associated with a comparable symptom burden and cardiac phenotype as in patients presenting later in childhood. Long-term outcomes including mortality did not differ by age of presentation, but patients presenting at younger than 12 years experienced adverse events at younger ages.

Although variants in cardiac sarcomeric protein genes are responsible for the majority of childhood-onset hypertrophic cardiomyopathy (HCM),^1^^,^^2^ because of variable and incomplete age-related penetrance, sarcomeric HCM has historically been considered a disease of adolescence or adulthood.^3^ However, recent childhood population studies have shown that up to 50% of children with sarcomeric HCM are diagnosed before 12 years of age,^4^, ^5^, ^6^ and longitudinal cohort studies have highlighted the significant morbidity and mortality associated with a childhood diagnosis of HCM.^4^, ^5^, ^6^, ^7^, ^8^ Despite this, the clinical presentation, natural history, and outcomes of preadolescent sarcomeric HCM have not been systematically characterized to date.

For decades, the management of HCM in both children and adults has focused on symptom palliation, family screening, and prevention of disease-related complications. With the advent of novel disease-modifying therapies such as myosin inhibitors^9^ and gene therapy programs,^10^ an improved understanding of age-specific differences would assist clinical care and could help guide the future use of disease-specific therapies in young children with HCM. The aim of this study was to describe the clinical characteristics and outcomes of a large, international, multicenter cohort of children with nonsyndromic HCM presenting before 12 years of age.

## Methods

### Study population

Children meeting diagnostic criteria for HCM between 1 and <12 years of age were identified from the International Paediatric Hypertrophic Cardiomyopathy Consortium (IPHCC), which contains a total cohort of 1,207 children with nonsyndromic disease diagnosed between 1970 and 2019.^11^ HCM was defined as a maximal left ventricular (LV) wall thickness >2 SDs higher than the body surface area–corrected population mean (*z*-score ≥+2).^12^ Supplemental Figure 1 describes participation, recruitment, and retention in the consortium. As the IPHCC cohort of patients was previously used to develop and validate the HCM Risk-Kids pediatric HCM risk prediction model for sudden cardiac death (SCD),^11^ it does not include patients with histories of resuscitated cardiac arrest, ventricular fibrillation, or sustained ventricular tachycardia (VT) prior to diagnosis. Patients with diagnoses of underlying inborn error of metabolism, RASopathy syndrome, or neuromuscular disease were excluded from this study. Patients diagnosed in infancy (<1 year) were excluded and have been previously described.^13^

### Data collection

Anonymized, noninvasive clinical data were collected from baseline evaluation and follow-up, including heart failure symptoms (New York heart Association or Ross functional classification^14^), family history, resting and ambulatory electrocardiography, transthoracic echocardiography (2-dimensional, Doppler, and color), and interventions (LV myectomy, implantable cardioverter-defibrillator [ICD] implantation). Heart failure symptoms were defined as New York Heart Association or Ross functional class ≥2. Maximal LV wall thickness and left atrial diameter measurements, obtained as previously described,^11^ are expressed in millimeters and *z*-scores relative to the distribution of measurements for body surface area in healthy children.^15^ LV outflow tract (LVOT) gradient was measured at rest. LVOT obstruction (LVOTO) was defined as an instantaneous peak Doppler LVOT pressure gradient ≥30 mm Hg.^12^ Moderate LVOTO was defined as a pressure gradient of 50-90 mm Hg and severe as a pressure gradient ≥90 mm Hg. Nonsustained VT was defined as ≥3 consecutive ventricular beats at a rate of >120 beats/min lasting <30 seconds on ambulatory electrocardiography.^12^ The indication for ICD implantation was defined as primary prevention in patients considered to be at high risk for life-threatening ventricular arrhythmia but who had not yet experienced documented cardiac arrest or sustained ventricular arrhythmia and as secondary prevention in patients who had already experienced cardiac arrest of sustained VT.^12^^,^^16^, ^17^, ^18^ Data were collected independently at each participating center, and data integrity is guaranteed by each author.

### Genetic testing

Genetic testing was performed at the discretion of the treating clinician as part of usual care. Variants reported in HCM disease-causing genes (sarcomeric and nonsarcomeric) were reclassified according to the American College of Medical Genetics and Genomics guidelines as pathogenic (P), likely pathogenic (LP), variant of unknown significance or likely benign/benign.^19^ Variants classified as P/LP were considered disease causing. Patients who did not undergo genetic testing were excluded from the analyses comparing patients with and without disease-causing variants identified on genetic testing.

### Outcomes

The primary study outcome was all-cause mortality or cardiac transplantation. The secondary outcome was first life-threatening arrhythmic event, defined as SCD or an equivalent event (resuscitated cardiac arrest, appropriate ICD therapy for ventricular tachyarrhythmia, or sustained VT associated with hemodynamic compromise). Outcomes were determined by the treating cardiologist at each center. Patients were classified as lost to follow-up if the last clinical review was >3 years before the study endpoint.

### Statistical analysis

The proportion of missing data is indicated for each variable. Continuous variables are described as mean ± SD or median (IQR) as appropriate, with 3-group comparisons conducted using analysis of variance or Wilcoxon rank sum tests, respectively. Categorical variables were compared using the chi-square test. Follow-up time was calculated from the date of first evaluation at a participating center to the date of reaching the study endpoint or the date of most recent evaluation prior to the end of the study (December 2019). The clinical characteristics of the cohort were compared with those of patients from the IPHCC cohort presenting in adolescence (>12 years), and estimates of survival by age of presentation (1-<12 years vs ≥12 years) were obtained using the Kaplan-Meier product limit method. A log-rank test was used to compare survival distributions between the 2 groups. The association of baseline clinical features with mortality or cardiac transplantation were assessed in a univariate and multivariate Cox proportional hazard model. A backward selection technique was used to identify variables that remained significant at the 0.1 level for inclusion in the multivariable model. Statistical analysis was performed using Stata version 15 (StataCorp).

### Ethics

This study conformed to the principles of the Declaration of Helsinki and Good Clinical Practice. Local ethics approval was given for each participating center with waiver of the requirement to obtain informed consent for retrospective, anonymized data. The data underlying this paper cannot be shared publicly, as consent for dissemination of patient data was not obtained.

## Results

Six hundred thirty-nine children (male, n = 417; 65.3%) were diagnosed between 1 and <12 years of age (median 7 years [IQR: 4-10 years]; 1-4 years, n = 199 [31.1%]; 5-8 years, n = 202 [31.6%]; 9-<12 years, n = 238 [37.2%]).

### Baseline clinical phenotype for those diagnosed between 1 and <12 years

The clinical phenotype at the time of baseline assessment is described in Table 1. One hundred forty-five patients (27.2%) had resting LVOTO, which was moderate in 62 (11.6%) and severe in 35 (6.6%). Of patients with heart failure symptoms, 45 (43.8%) had LVOTO at baseline. Two hundred thirty-eight (37.4%) were receiving, or commenced on, beta-blockers at baseline assessment at their respective IPHCC centers. A comparison of the baseline clinical phenotype for the subgroup of patients presenting at younger than 6 years is shown in Supplemental Table 1.

**Table 1:** Clinical Characteristics and Natural History of Preadolescent and Adolescent Nonsyndromic Hypertrophic Cardiomyopathy

	1-<12 y (n = 639)		≥12 y (n = 568)		
		Missing Data		Missing Data	*P* Value
Median age at presentation, y	7 (4-10)	—	14 (13-15)		<0.001
Male	417 (65.3)	—	398 (70.1)	—	0.107
Family history of HCM	339 (53.6)	7 (1.1)	299 (53.6)	10 (1.8)	0.264
Family history of SCD	67 (10.5)	—	81 (14.3)	—	0.046
Unexplained syncope	39 (6.1)	—	70 (12.3)	1 (0.2)	<0.001
NYHA/Ross functional class >I	132 (20.9)	8 (1.3)	137 (24.6)	10 (1.8)	0.149
Beta-blockers	238 (37.4)	2 (0.3)	231 (40.7)	1 (0.2)	0.673
Baseline clinical investigations					
NSVT	25 (4.9)	127 (19.9)	31 (6.1)	61 (10.7)	0.109
LVMWT, mm	13.6 (10-19)	23 (3.6)	17 (13-24)	10 (1.8)	<0.001
LVMWT *z*-score	8.7 (5.3-14.4)	72 (11.3)	8.9 (5.8-15.5)	32 (5.6)	0.129
LA diameter, mm	28.9 ± 8.5	203 (31.8)	35.5 ± 8.6	117 (20.6)	<0.001
LA diameter *z*-score	1.2 (0.13-2.86)	230 (36.0)	1.5 (0.19-3.0)	130 (22.9)	0.4111
LVOT gradient, mm Hg	10 (6-32)	105 (16.4)	8 (5-16)	60 (10.6)	0.0005
LVOT obstruction	145 (27.2)	105 (16.4)	82 (16.1)	60 (10.6)	<0.001
Severe LVOT obstruction	35 (6.6)	105 (16.4)	18 (3.5)	60 (10.6)	0.027
Clinical follow-up and outcomes					
Length of follow-up, y	5.6 (2.3-10)	4 (0.6)	4.4 (2.4-8.5)	1 (0.2)	0.018
Myectomy	67 (10.5)	3 (0.5)	41 (7.2)	1 (0.2)	0.045
ICD implantation	148 (23.3)	4 (0.6)	184 (32.7)	5 (0.9)	<0.001
ICD indication		6 (4.1)		4 (2.2)	
Primary	121 (81.8)	—	170 (92.4)	—	0.005
Secondary	21 (14.2)	—	10 (5.4)	—	
Pacemaker	21 (3.4)	24 (3.8)	33 (5.9)	11 (1.9)	0.041
Pacemaker indication		2 (9.5)		5 (15.2)	
Sinoatrial disease	5 (23.8)	—	2 (6.1)	—	0.147
AV node disease	6 (28.6)	—	8 (24.2)	—	
LVOT obstruction	8 (38.1)	—	18 (54.6)	—	
Death or cardiac transplantation	63 (9.9)	—	45 (7.9)	—	0.771
SCD	31 (4.9)	—	22 (3.9)	—	0.282
Heart failure	5 (0.8)	—	2 (0.4)	—	
Other CV	3 (0.5)	—	3 (0.6)	—	
Non-CV	1 (0.2)	—	2 (0.4)	—	
Unknown death	2 (0.3)	—	6 (1.1)	—	
Transplantation	21 (3.3)	—	10 (1.8)	—	
Time to death/transplantation from presentation, y	5.5 (2.6-8.8)	—	3.3 (1.6-7.3)	—	0.117
Life-threatening arrhythmic event	69 (10.8)	—	61 (36.1)	—	0.974
SCD	31 (44.9)	—	22 (36.1)	—	0.104
Resuscitated arrest	17 (24.6)	—	9 (14.8)	—	
Appropriate ICD therapy	14 (20.3)	—	24 (39.3)	—	
Sustained VT	7 (10.2)	—	6 (9.8)	—	
Time to life-threatening arrhythmia from presentation, y	4.8 (2.1-8.8)		3.0 (1.6-6.3)		0.1080

Values are median (IQR), n (%), or mean ± SD.

AV = atrioventricular; CV = cardiovascular; HCM = hypertrophic cardiomyopathy; ICD = implantable cardioverter-defibrillator; LA = left atrial; LVMWT = left ventricular maximal wall thickness; LVOT = left ventricular outflow tract; NSVT = nonsustained ventricular tachycardia; NYHA = New York Heart Association; SCD = sudden cardiac death; VT = ventricular tachycardia.

### Genetic testing for those diagnosed between 1 and <12 years

The genetic testing strategy is shown in Supplemental Figure 2. In brief, genetic testing information was available for 528 patients (82.6%), of whom 348 (65.9%) had undergone genetic testing. Genetic testing status was therefore unknown or not performed in 291 patients (45.5%). Patients who underwent genetic testing did not significantly differ in baseline characteristics compared with those without genetic testing (Supplemental Table 2). Following American College of Medical Genetics and Genomics reclassification of variants previously classified as disease causing (n = 253), 186 patients (53.5%) had P/LP variants, 61 (17.5%) had variants of unknown significance, and 6 (1.7%) had likely benign/benign variants. Genetic testing was negative or identified only likely benign/benign variants in 76 patients (21.8%). Disease-causing variants (P/LP) were most commonly identified in *MYH7* (n = 76 [43.4%]) or *MYBPC3* (n = 65 [37.1%]) and are described in Table 2. Eleven patients (5.9%) were compound heterozygous or homozygous for disease-causing variants (Table 2, Supplemental Table 3). Eleven patients had variants in nonsarcomeric genes that were considered disease causing (P/LP): *JPH2* (n = 4), *PRKAG2* (n = 2), Desmin (n = 2), *FHOD3* (n = 1), *KRAS* (n = 1), and *RAF1* (n = 1). Patients with P/LP variants were more likely to have family histories of HCM (n = 131 [71.2%] vs n = 54 [40.9%]; *P* < 0.001) but did not otherwise differ in baseline characteristics (Supplemental Tables 4 and 5).

**Table 2:** Disease-Causing Variants Identified on Genetic Testing in Preadolescent and Adolescent Hypertrophic Cardiomyopathy

	1-<12 y (n = 186)	≥12 y (n = 151)
Single variant		
Thick filament	139 (74.7)	109 (72.2)
*MYBPC3*	64	59
*MYH7*	75	50
Thin filament	25 (13.4)	33 (21.9)
*ACTC*	5	4
*MYL2*	4	6
*MYL3*	1	0
*TPM1*	7	8
*TNNI3*	4	8
*TNNC1*	1	0
*TNNT2*	3	6
*FLNC*	0	1
Nonsarcomeric	11 (5.9)	4 (2.6)
*JPH2*	4	0
*KRAS*	1	0
*PRKAG2*	2	2
*RAF1*	1	1
*DES*	2	1
*FHOD3*	1	0
≥2 variants		
*MYBPC3* (P) + *TPM1* (P)	1	
*MYBPC3* (P) + *MYH7* (LP)	2	1
*MYBPC3* (P) + *MYBPC3* (P)	1	1
*MYH7* (P) + *MYH7* (LP)		1
*DES* (P) + *MYH7* (LP)	1	
*MYBPC3* (P) + *MYBPC3* (LP)	2	
*MYBPC3* (P) + *TNNT2* (P)		1
*MYH7* (P) + *TNNT2* (P)	2	
*MYBPC3* (P) + *MYH7* (P)	1	
*MYBPC3* (P) + *DMD* (LP)	1	
*MYH7* (LP) + *MYH7* (LP)		1

Values are n (%) or n.

LP = likely pathogenic; P = pathogenic.

### Outcomes in patients diagnosed between 1 and <12 years

**Figure 1:**
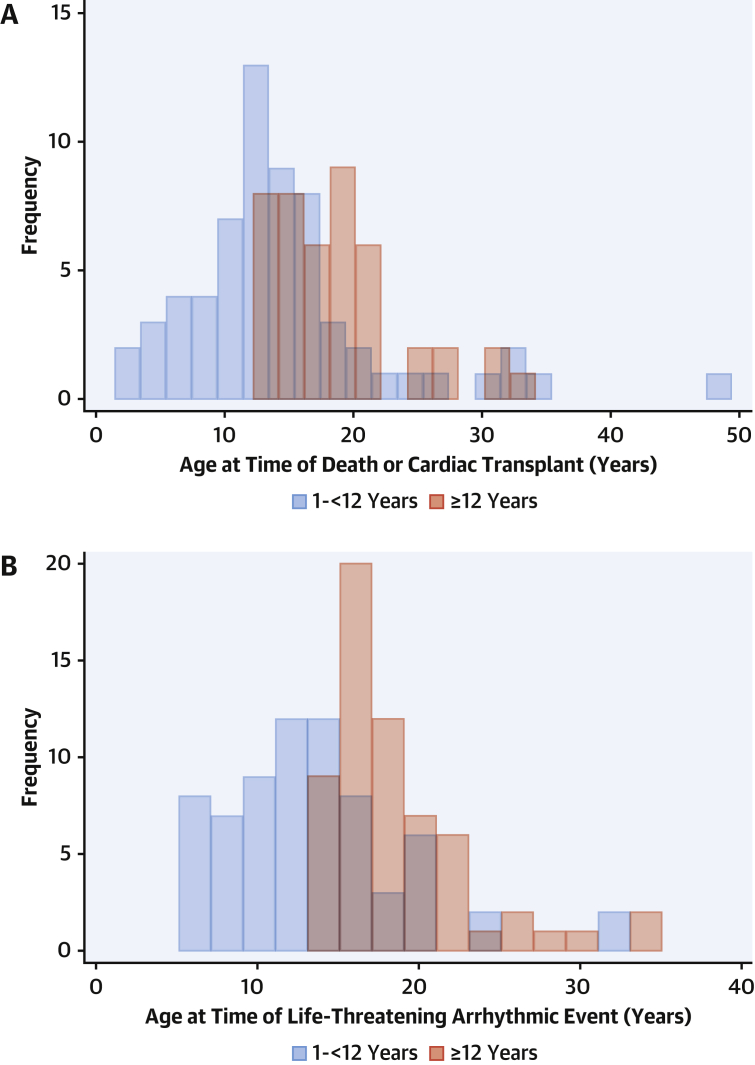
Age at Time of Death or Transplantation and Life-Threatening Arrhythmic Events Patients presenting at 1-<12 years of age experienced adverse events at a younger age: **(A)** death or cardiac transplantation and **(B)** life-threatening arrhythmic events.

**Figure 2:**
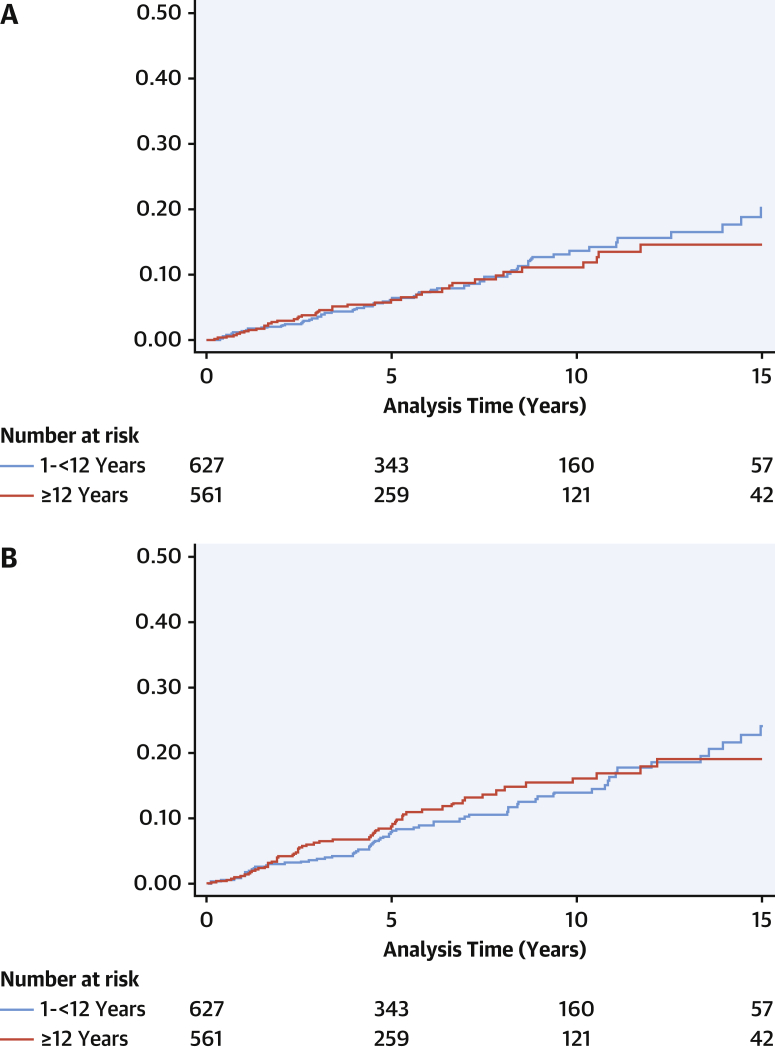
Cumulative Incidence of Death or Transplantation and Life-Threatening Arrhythmic Events The cumulative incidence was similar in those presenting at 1-<12 years of age and ≥12 years of age for **(A)** death or cardiac transplantation (*P* = 0.447) and **(B)** life-threatening arrhythmic events (*P* = 0.104).

The median length of follow-up was 5.6 years (IQR: 2.3-10 years), during which 67 patients (10.5%) underwent LV septal myectomy, 21 (3.4%) required pacemaker implantation, and 148 (23.3%) underwent ICD implantation for primary (n = 121 [81.8%]) or secondary (n = 21 [14.2%]) prevention. Two patients required pacemakers following LV septal myectomy for postoperative atrioventricular node block. Forty-two patients (6.6%) died, of which 31 deaths (4.9%) were due to SCD, 5 (0.8%) to heart failure, 3 (0.5%) to other cardiovascular causes, 1 (0.2%) to a noncardiovascular cause, and 2 (0.3%) to unknown causes. Twenty-one patients (3.3%) underwent cardiac transplantation. The median age at the time of death or cardiac transplantation was 12.9 years (IQR: 10.3-16.5 years; range: 1.0-49.2 years) (Figure 2A). The overall annual incidence of mortality or cardiac transplantation was 1.41% (95% CI: 1.10%-1.81%) (Figure 1A). On multivariable analysis, the presence of heart failure symptoms, increasing left atrial diameter *z*-score, and the absence of a disease-causing variant on genetic testing were associated with reaching the mortality or transplantation endpoint (Supplemental Table 6). Sixty-nine patients (10.8%) experienced life-threatening arrhythmic events: SCD (n = 31 [4.9%]), resuscitated cardiac arrest (n = 17 [2.7%]), appropriate ICD therapy (n = 14 [2.2%]), or sustained VT with hemodynamic compromise (n = 7 [1.1%]). The median age at the time of life-threatening arrhythmic event was 12.6 years (IQR: 9.8-15.7 years; range: 5.1-32.7 years) (Figure 2B), and no patients experienced events before 5 years of age. Of these, 23 patients (33.3%) had previously undergone implantation of primary prevention ICDs. Three patients experienced sudden death with ICDs in situ, but electrograms and information on cardiac rhythm at the time of death were not available for review. The overall annual incidence of life-threatening arrhythmic events was 1.52% (95% CI: 1.20%-1.93%) (Figure 1B).

### Comparison of HCM phenotype and outcomes by age of presentation

Table 1 compares the clinical characteristics and outcomes of this cohort with 568 patients presenting after 12 years. Those presenting between 1 and <12 years were less likely to have family histories of SCD (n = 67 [10.5%] vs n = 81 [14.3%]; *P* = 0.046) or to report unexplained syncope (n = 39 [6.1%] vs n = 70 [12.3%]; *P* < 0.001). The degree of LV hypertrophy at baseline did not differ, but a higher proportion of patients aged 1-<12 years had LVOTO (n = 145 [27.2%] vs n = 82 [16.1%]; *P* < 0.001). During follow-up, a higher proportion of patients aged 1-<12 years underwent myectomy (n = 67 [10.5%] vs n = 41 [7.2%]; *P* = 0.045) but a lower proportion received primary prevention ICDs (n = 121 [18.9%] vs n = 171 [30.1%]; *P* = 0.041). Five-year transplantation-free survival (93.6% [95% CI: 90.9%-95.5%] in those 1-<12 years of age vs 93.9% [95% CI: 97.1%-99.2%] in those ≥12 years of age; *P* = 0.750) and cause of death did not differ between the 2 groups (Central Illustration). Time from presentation to death or cardiac transplantation did not differ by age group, meaning that those presenting before 12 years of age had a younger median age of death or cardiac transplantation (12.9 vs 18.3 years; *P* < 0.001) (Figure 2A).

**Central Illustration:**
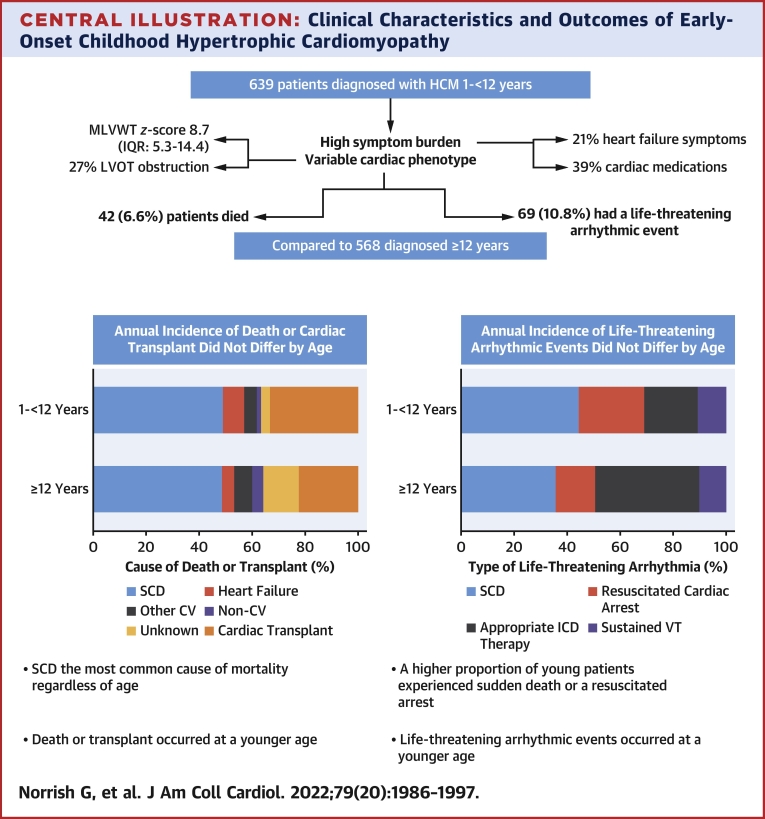
Clinical Characteristics and Outcomes of Early-Onset Childhood Hypertrophic Cardiomyopathy Children presenting at 1-<12 years of age have comparable outcomes as those presenting later in childhood but experience adverse events (death, cardiac transplantation, or life-threatening arrhythmias) at a younger age. CV = cardiovascular; LVOT = left ventricular outflow tract; MLVWT = maximal left ventricular wall thickness.

The annual incidence of life-threatening arrhythmic events did not differ between the groups (1.52 [95% CI: 1.20-1.93] vs 1.76 [95% CI: 1.27-2.26]; *P* = 0.447). SCD and resuscitated cardiac arrest were more frequent in younger patients (4.7% vs 3.9% and 2.7% vs 1.6%, respectively), but this was not statistically significant (*P* = 0.104) (Figure 2B). Time from diagnosis to a life-threatening arrhythmic event did not differ significantly by age of presentation, meaning that those presenting before 12 years of age experienced events at a younger median age (13.9 years vs 18.7 years; *P* < 0.001).

## Discussion

To our knowledge, this study is the first systematic description of early childhood–onset HCM. The findings highlight that young patients have a comparable symptom burden and long-term outcomes as those presenting later in childhood (Central Illustration), with the implication that even the youngest children with HCM may benefit from similar management strategies and novel therapeutic approaches as used in older patients with HCM.

### Phenotype of preadolescent HCM

Sarcomeric HCM has historically been considered a disease of adolescence or early adulthood, yet more recent studies have suggested that disease penetrance during childhood may be higher than previously recognized.^20^, ^21^, ^22^ Nonetheless, it remains a rare disease, with an estimated overall incidence of <0.5 per 100,000 live births.^23^, ^24^, ^25^ The phenotype of nonsyndromic childhood HCM is recognized to be highly variable, but whether age at presentation influences disease severity has not been previously investigated.^4^^,^^6^^,^^8^^,^^26^ In this study, one-fifth of children with preadolescent childhood HCM were symptomatic at baseline, and the cardiac phenotype was variable, including severe hypertrophy, LVOTO, and life-threatening ventricular arrhythmias occurring during follow-up. This is comparable with previous population studies with unselected childhood disease.^7^^,^^27^^,^^28^ Although the proportion of patients with heart failure symptoms did not differ by age, younger patients were less likely to report unexplained syncope. This could be due to difficulties in distinguishing the cause of syncopal events in young children. However, it may also suggest age-related differences in patient-reported symptoms not explained by differences in phenotype. The degree of hypertrophy was not significantly different between the 2 groups, but a higher proportion of preadolescent patients had LVOTO, and an important minority (6%) had severe obstruction (gradient ≥90 mm Hg). We speculate that this could be explained by undiagnosed nonsarcomeric disease, which is more commonly associated with LVOTO and typically presents at a younger age.^29^ However, as the difference persisted in subgroup analysis of patients with identified sarcomeric disease-causing variants, it is more likely that age-related differences in phenotype expression of sarcomeric disease exist. The progression or development of LVOTO in childhood disease is poorly understood, and future studies using serial clinical data are required to investigate the changing burden of LVOTO during childhood, as well as the effect of surgical and pharmacologic septal reduction strategies on prognosis.

### Genetics of preadolescent HCM

Recent data have suggested a higher yield of genetic testing for sarcomere protein gene mutations in childhood-onset HCM (1-18 years of age) compared with adult cohorts.^5^ Genetic testing was not performed systematically in our cohort, and it was therefore beyond the scope of this study to investigate the yield of genetic testing. Nonetheless, one strength of this study is that genetic testing information was available for more than one-half of the cohort, identifying disease-causing variants in more than 50%. This suggests that the yield of genetic testing in preadolescent disease is similarly high and confirms the contribution of sarcomeric disease to early childhood–onset disease.^1^^,^^2^^,^^30^ A disease-causing variant was more likely to be identified if there was a family history of HCM at baseline, as has been reported in previous adult and childhood cohorts.^5^^,^^31^, ^32^, ^33^ In agreement with previous childhood reports, P/LP variants were most commonly identified in *MYH7* and *MYBPC3*.^5^ Although compound heterozygote or homozygote sarcomeric variants were more common in the preadolescent group, this remained relatively rare (6%), which suggests that the majority of HCM presenting in childhood is caused by single high-impact gene variants. A small number of patients had disease-causing variants identified in nonsarcomeric protein genes. This could suggest undiagnosed syndromic disease but may also support the role of nonsarcomeric variants contributing to disease phenotype in children with clinically nonsyndromic disease.^34^

### Impact of age on the outcomes of preadolescent HCM

Outcomes during childhood for most patients with childhood-onset HCM are good,^4^^,^^6^^,^^27^ but recent data from SHaRE (Sarcomeric Human Cardiomyopathy Registry) have highlighted the significant morbidity and mortality associated with early-onset disease, with more than one-fifth experiencing adverse cardiac events within 10 years of diagnosis.^4^, ^5^, ^6^^,^^26^^,^^35^ In this study, the incidence of death or cardiac transplantation was similar for those presenting before 12 years of age and in later childhood, but for those presenting earlier in childhood, these events occurred at a younger age, and three-quarters took place before 20 years of age. Increasing age at presentation was associated with a higher risk for mortality or cardiac transplantation on univariate analysis, but this did not remain significant on multivariate analysis. The causes of death were similar in both groups, with SCD remaining the most frequent cause regardless of age of presentation, but a higher proportion of preadolescent patients underwent cardiac transplantation. This is in contrast to a recent North American study, which found that non-SCD death and transplantation occurred more frequently in those diagnosed before 5 years of age, although the majority of these events occurred in infancy, an age range excluded from this study.^27^

Children are recognized to have a higher incidence of arrhythmic events compared with adult patients, but the relationship between age and risk remains unclear.^8^^,^^11^^,^^27^^,^^36^^,^^37^ In this study, the overall incidence of arrhythmic events was similar for those presenting prior to or during adolescence, but there was a trend toward a higher prevalence of SCD and resuscitated cardiac arrest rather than appropriate ICD therapies in the younger age group. Patients presenting before 12 years of age were less likely to undergo ICD implantation during follow-up but were more likely to require ICD implantation for secondary prophylaxis following a life-threatening arrhythmia. This could suggest a difference in clinicians’ perception of risk and subsequent management for those presenting at a younger age, as well as reflecting technical difficulties in implanting ICDs in small patients.^38^ Of note, no patients experienced events before 5 years of age, which could suggest that the very young patients, in whom device implantation may represent a technical challenge, may be less likely to benefit from ICD implantation. The time to a life-threatening arrhythmic event did not differ by age of presentation, and arrhythmic events occurred at a younger age in those presenting in preadolescence. This is in contrast to findings reported by Miron et al,^27^ in which the frequency of arrhythmic events was highest during adolescent years regardless of age of diagnosis. Our understanding of the interaction among age at presentation, current age, and risk stratification remains incomplete, and recently published pediatric risk models have differed in their treatment of age at presentation as a risk factor for arrhythmic events.^11^^,^^27^ In previous analysis of this dataset, including age as a predictor variable did not improve the ability to predict arrhythmic events (HCM Risk-Kids).^11^ In contrast, an alternative North American risk model reported that increasing age was associated with an increasing risk for arrhythmic events.^27^ Further exploration of the relationship is needed, but the data from this study highlight that all childhood patients are at risk for arrhythmic events and stress the importance of risk stratification as a cornerstone of clinical care.

### Study limitations

This study had inherent limitations due to its multicenter and retrospective design, including missing data and incomplete recruitment of eligible patients. Variations in clinical assessment and patient management are inevitable, as patients were recruited from multiple centers and different geographic locations. Previous analysis of this cohort, however, has shown no difference in outcomes by era of presentation, which may be explained by no significant change in management strategies over time, with the exception of ICD implantation.^4^^,^^11^ A diagnosis of HCM may be made following presentation with symptoms, incidentally or through family screening, with the implication that the timing of HCM diagnosis may be significantly determined by local health care screening patterns.^4^ It is plausible that symptomatic patients, who may have phenotypically more severe disease and worse outcomes, could present at a younger age, introducing bias to the analysis. Data on reason for presentation were not collected in this cohort, although a similar proportion were symptomatic (heart failure symptoms or syncope) at baseline and had family histories of HCM in both age groups. The exclusion of patients presenting with out-of-hospital ventricular fibrillation arrest could have further biased the results toward patients with a less severe disease phenotype. However, a previous national cohort study of childhood HCM from United Kingdom has reported that this is a rare (<4%) reason for presentation during childhood.^4^ As sudden death is a rare event, a composite definition of life-threatening arrhythmias was used for this study, which includes appropriate ICD therapies. This is in keeping with previous studies in adults and children with HCM,^11^^,^^27^^,^^36^^,^^39^ but it is plausible that not all appropriate therapies would have necessarily resulted in sudden death untreated. Genetic testing was not performed systematically in this cohort, therefore it is beyond the scope of this study to investigate the yield of genetic testing in preadolescent nonsyndromic HCM.

## Conclusions

In this retrospective study, childhood HCM presenting before 12 years of age was associated with a comparable symptom burden and cardiac phenotype as in patients presenting later in adolescence. Long-term outcomes including mortality did not differ by age of presentation, but those presenting before 12 years of age experienced adverse events at a younger age. Patients diagnosed before 12 years of age experienced life-threatening arrhythmias at the same rate as those presenting during adolescence but were less likely to receive primary prevention ICDs. This study supports the notion that preadolescent patients should not be considered a distinct entity for risk stratification and that similar management strategies as used in older patients with HCM should be used.

Perspectives **COMPETENCY IN PATIENT CARE AND PROCEDURAL SKILLS:** Young children with HCM have a phenotype similar to patients presenting later and are often symptomatic, warranting early therapeutic intervention.

**TRANSLATIONAL OUTLOOK:** Future studies address the impact of patient age on the risk for arrhythmias and other adverse events associated with HCM and evaluate the benefit of disease-modifying therapies in children.

## Funding Support and Author Disclosures

This work was supported by the British Heart Foundation (grant FS/16/72/32270) to Drs Norrish and Kaski. This work is (partly) funded by the National Institute for Health Research Great Ormond Street Hospital Biomedical Research Centre. Dr Norrish is supported by Great Ormond Street Hospital Children’s Charity. Drs Field and Kaski are supported by Max’s Foundation and Great Ormond Street Hospital Children’s Charity. Dr Kaski is supported by a Medical Research Council–National Institute for Health Research Clinical Academic Research Partnership award. This work was financially supported by the Foundation for Paediatric Research of Finland (Dr Ojala). Dr Fernandez has received speaker fees from Sanofi-Genzyme. Dr Kubus is supported by MH CZ – DRO, Motol University Hospital (00064203). All other authors have reported that they have no relationships relevant to the contents of this paper to disclose.
