# Supplementary material for: Clinical Features and Natural History of Preadolescent Nonsyndromic Hypertrophic Cardiomyopathy
Source: J Am Coll Cardiol. 2022 May 24;79(20):1986–97. doi: 10.1016/j.jacc.2022.03.347 (PMC9125690; doi:10.1016/j.jacc.2022.03.347)
Supplement: Supplemental Tables 1–6 and Figures 1 and 2 [file mmc1.docx]

**Title: Clinical Features and Natural History of Preadolescent Nonsyndromic Hypertrophic Cardiomyopathy**

**Brief Title: Preadolescent Nonsyndromic Hypertrophic Cardiomyopathy**

Gabrielle Norrish (1,2) BMBCh, Aoife Cleary (1), Ella Field (1) BA, Elena Cervi (1) MD, Olga Boleti (2) MD, Lidia Ziółkowska (4) MD, Iacopo Olivotto (5) MD, Diala Khraiche (6) MD, Giuseppe Limongelli (7) MD, Aris Anastasakis (8) MD, Robert Weintraub (9) MD, Elena Biagini (10) MD, Luca Ragni (10) MD , Terence Prendiville (11) MD , Sophie Duignan (11) MD, Karen McLeod (12) MD, Maria Ilina (12) MD, Adrian Fernandez (13) MD, Chiara Marrone (14,15) MD, Regina Bökenkamp (16) MD, Anwar Baban (17) MD, Peter Kubus (18) MD, Piers.E.F Daubeney (19) MD, Georgia Sarquella-Brugada (20) MD, Sergi Cesar (20) MD, Sabine Klaassen (21,22,23) MD, Tiina H Ojala (24) MD, Vinay Bhole (25) MD, Constancio Medrano (26) MD, Orhan Uzun (27) MD, Elspeth Brown (28) MD, Ferran Gran (29) MD, Gianfranco Sinagra (30) MD, Francisco J Castro (31) MD, Graham Stuart (32) MD, Hirokuni Yamazawa (33) MD, Roberto Barriales-Villa (34) MD, Luis Garcia-Guereta (35) MD, Satish Adwani (36) MD, Katie Linter (37) MD, Tara Bharucha (38) MD, Esther Gonzales-Lopez(39) MD, Ana Siles (39) MD, Torsten B Rasmussen (40) MD, Margherita Calcagnino (41) MD, Caroline B Jones (42) MD, Hans De Wilde (43) MD, Toru Kubo (44) MD, Tiziana Felice (45) MD, Anca Popoiu (46) MD, Jens Mogensen (47) MD, Sujeev Mathur (48) MD, Fernando Centeno (49) MD, Zdenka Reinhardt (50) MD, Sylvie Schouvey (51) MD, Perry M Elliott (2,52) MD, Juan Pablo Kaski (1,2) MD

|  | | | | **< 6 years (n= 242)** | | **Missing data** | | **6-12 years (n= 397)** | | **Missing data** | | **P value** | |
| --- | --- | --- | --- | --- | --- | --- | --- | --- | --- | --- | --- | --- | --- |
|  | | | |  | |  | |  | |  | |  | |
| Male sex | | | | 156 (64.5%) | | - | | 261 (65.7%) | | - | | 0.742 | |
| FHx HCM | | | | 111 (46.1%) | | 1 (0.00%) | | 228 (58.3%) | | 6 (1.5%) | | 0.010 | |
| FHx SCD | | | | 20 (8.3%) | | - | | 47 (11.8%) | | - | | 0.153 | |
| Unexplained syncope | | | | 13 (5.4%) | | - | | 26 (6.6%) | | - | | 0.547 | |
| NYHA/Ross>1 | | | | 35 (14.5%) | | 3 (0.01%) | | 97 (24.4%) | | 5 (1.3%) | | 0.003 | |
| B Blockers | | | | 87 (36.3%) | | 2 (0.01%) | | 151 (38.0%) | | - | | 0.757 | |
| NSVT | | | | 8 (4.2%) | | 61 (25.2%) | | 17 (5.0%) | | 66 (16.7%) | | 0.337 | |
| Z score LVMWT [median (IQR)] | | | | 8.3 (5.1, 13.7) | | 34 (14.0%) | | 8.9 (5.5, 14.9) | | 38 (9.6%) | | 0.2022 | |
| Z score LA [median (IQR)] | | | | 1.2 (-0.1-2.6) | | 109 (45.0%) | | 1.2 (0.2 – 3.0) | | 121 (30.5%) | | 0.0012 | |
| LVOT gradient [median (IQR)] | | | | 10 (5, 46) | | 45 (18.6%) | | 10 (6,25) | | 60 (15.1%) | | 0.4312 | |
| LVOT obstruction | | | | 63 (32.0%) | | 45 (18.6%) | | 82 (24.3%) | | 60 (15.1%) | | 0.055 | |
| Myectomy | | | | 35 (14.5%) | | - | | 32 (8.1%) | | 3 (0.01%) | | 0.011 | |
| ICD implantation | | | | 48 (19.8%) | | - | | 100 (25.5%) | | 4 (0.01%) | | 0.104 | |
|  | | Primary | | 34 (79.1%) | | - | | 87 (87.9%) | | - | | 0.174 | |
|  |  | Secondary | | 9 (20.9%) | | - | | 12 (12.1%) | | - | |  |  |
| Death or cardiac transplant | | | | 19 (7.9%) | | - | | 44 (11.1%) | | - | | 0.291 | |
|  | | SCD | | 10 (4.1%) | | - | | 21 (5.3%) | | - | |  | |
|  |  | Heart failure | | 4 (1.7%) | | - | | 1 (0.3%) | | - | |  |  |
|  |  | Other-CV | | 0 (0.0%) | | - | | 3 (0.7%) | | - | |  |  |
|  |  | Non- CV | | 1 (0.4%) | | - | | 0 (0.0%) | | - | |  |  |
|  |  | Unknown death | | 0 (0.0%) | | - | | 2 (0.5%) | | - | |  |  |
|  |  | Transplant | | 4 (1.7%) | | - | | 17 (4.3%) | | - | |  |  |
| Life threatening arrhythmic event | | | 23 (9.5%) | | - | | 46 (11.6%) | | - | | 0.411 | |  |
|  | SCD | | 10 (4.1%) | | - | | 21 (5.3%) | | - | |  | |  |
|  | Resuscitated arrest | | 9 (3.7%) | | - | | 8 (2.0%) | | - | |  |  |  |
|  | Appropriate ICD therapy | | 1 (0.4%) | | - | | 13 (3.3%) | | - | |  |  |  |
|  | Sustained VT | | 3 (1.2%) | | - | | 4 (1.0%) | | - | |  |  |  |

Supplementary table 1: Comparing the clinical characteristics and natural history of patients presenting under and over the age of 6 years.

HCM = hypertrophic cardiomyopathy, SCD = sudden cardiac death, NYHA = New York heart association, NSVT = non-sustained ventricular tachycardia, LVMWT = left ventricular maximal wall thickness, SD = standard deviation, IQR = interquartile range, LA = left atrial, LVOT = left ventricular outflow tract, ICD = implantable cardiac defibrillator, AV = atrioventricular, CV = cardiovascular, SCD = sudden cardiac death, VT = ventricular tachycardia

|  | **Pre-adolescent** | | | **Adolescent** | | |
| --- | --- | --- | --- | --- | --- | --- |
|  | Genetic testing (n=348) | No genetic testing (n=180) | P value | Genetic testing (n=287) | No genetic testing (n=178) | P value |
| Age at baseline | 6.5 (3.4) | 6.5 (3.5) | 0.9872 | 13.9 (1.3) | 13.9 (.2) | 0.7586 |
| Male sex | 226 (64.9) | 116 (64.4) | 0.715 | 200 (69.9) | 130 (73.0) | 0.473 |
| FHx HCM | 203 (59.0) | 88 (49.4) | 0.177 | 161 (56.9) | 86 (49.4) | 0.120 |
| FHx SCD | 38 (10.9) | 17 (9.4) | 0.515 | 48 (16.7) | 17 (9.6) | 0.030 |
| Unexplained syncope | 21 (6.0) | 12 (6.7) | 0.711 | 37 (12.9) | 22 (12.4) | 0.884 |
| NYHA/Ross>1 | 63 (18.3) | 49 (27.2) | 0.036 | 62 (21.6) | 42 (23.6) | 0.616 |
| NSVT | 14 (4.8) | 9 (5.8) | 0.918 | 13 (4.8) | 13 (7.9) | 0.349 |
| B Blockers | 126 (36.3) | 75 (41.7) | 0.440 | 123 (42.9) | 78 (43.8) | 0.124 |
| Z score LVMWT | 10.3 (7.2) | 10.4 (7.2) | 0.8546 | 11.7 (6.8) | 9.8 (7.3) | 0.0045 |
| Z score LA | -0.8 (4.6) | -0.9 (5.3) | 0.8440 | -0.5 (4.2) | 0.5 (4.6) | 0.0289 |
| LVOT gradient | 10 (5,30) | 10 (5.8,30) | 0.6240 | 9 (5,17) | 8 (5,15) | 0.2751 |
| Death | 9 (2.6%) | 23 (12.8) | <0.001 | 9 (3.1) | 8 (4.5) | 0.484 |
| Life threatening arrhythmic event | 39 (11.2) | 21 (11.7) | 0.555 | 29 (10.1) | 18 (10.1) | 0.998 |

Supplementary table 2: Comparing the clinical characteristics and natural history of patients with and without genetic testing

HCM = hypertrophic cardiomyopathy, SCD = sudden cardiac death, NYHA = New York heart association, NSVT = non-sustained ventricular tachycardia, LVMWT = left ventricular maximal wall thickness, SD = standard deviation, IQR = interquartile range, LA = left atrial, LVOT = left ventricular outflow tract,

| Patient | Gene | Protein/nucleotide change | ACMG classification |
| --- | --- | --- | --- |
| 1 | MYH7 | p.Arg869His  c.2606G>A | Likely pathogenic |
|  | MYBPC3 | p.Lys1065Glnfs*12  c.3192dup | Pathogenic |
| 2 | MYBPC3 | p.Trp1078*  c.3234G>A | Pathogenic |
|  | TPM1 | p.Met281Val  c.841A>G | Pathogenic |
| 3 | MYBPC3 | p.Val219Leu  c.655G>C | Pathogenic |
|  | MYH7 | p.Val606Met  c.1816G>A | Pathogenic |
| 4 | MYH7 | p.Asp239Asn  c.715G>A | Likely pathogenic |
|  | TNNT2 | p.Arg285Cys  c.853C>T | Pathogenic |
| 5 | MYH7 | p.Ala 355Thr  c.1063G>A | Pathogenic |
|  | TNNT2 | p.Asn281Ile  c.842A>T | Likely Pathogenic |
| 6 | MYBPC3 | p.Arg502Trp  c.1504C>T | Pathogenic |
|  | MYBPC3 | c.1624+4A>T | Pathogenic |
| 7 | MYBPC3 | p.Arg845Cys  c.2533C>Y | Likely pathogenic |
|  | MYBPC3 | c.3330+5G>c | Pathogenic |
| 8 | MYBCP3 | p.Lys1065fsX1076  c.3191insC | Pathogenic |
|  | MYH7 | p.Glu930Gln  c.2788G>C | Likely pathogenic |
| 9 | MYBPC3 | c.2308+1G>A | Pathogenic |
|  | MYBPC3 | p.75D>N | Likely pathogenic |

Supplementary table 3: Genetic variants of patients with compound heterozygous or homozygous sarcomeric variants.

|  | **1-<12 years** | | | **>=12 years** | | |
| --- | --- | --- | --- | --- | --- | --- |
|  | Disease causing variant (P/LP)  (n=186) | No disease causing variant  (n=162) | P value | Disease causing variant (P/LP)  (n=134) | No disease causing variant  (n=152) | P value |
| Age at baseline | 6.5 (3.4) | 6.5 (3.3) | 0.4465 | 13.9 (1.4) | 13.9 (1.2) | 0.4662 |
| Male sex | 114 (61.3) | 112 (69.1) | 0.126 | 101(66.5) | 99 (73.9) | 0.171 |
| FHx HCM | 131 (71.2) | 72 (45.0) | <0.001 | 105 (70.0) | 56 (42.11) | <0.001 |
| FHx SCD | 21 (11.3) | 17 (10.5) | 0.812 | 31 (20.4) | 17 (12.6) | 0.077 |
| Unexplained syncope | 12 (6.5) | 9 (5.6) | 0.726 | 19 (12.5) | 18 (13.3) | 0.833 |
| NYHA/Ross>1 | 29 (15.6) | 34 (21.0) | 0.192 | 29 (19.1) | 33 (24.4) | 0.270 |
| NSVT | 7 (4.6) | 7 (5.1) | 0.863 | 6 (4.2) | 7 (5.6) | 0.682 |
| B Blockers | 62 (33.5) | 64 (39.5) | 0.263 | 60 (39.5) | 63 (46.7) | 0.249 |
| Z score LVMWT | 10.6 (7.5) | 9.9 (6.7) | 0.8139 | 12.6 (7.0) | 10.7 (6.6) | 0.9897 |
| Z score LA | -0.4 (4.3) | -1.3 (4.9) | 0.9560 | -0.2 (4.0) | -0.7 (4.4) | 0.8131 |
| LVOT gradient | 9 (6,20) | 10 (5,35) | 0.2768 | 8 (5,15) | 10 (5, 21) | 0.1526 |
| LVOT obstruction | 32 (20.9) | 42 (30.4) | 0.063 | 24 (17.0) | 25 (21.0) | 0.413 |
| Death | 4 (2.2) | 5 (3.1) | 0.583 | 3 (2.0) | 6 (4.4) | 0.231 |
| Life threatening arrhythmic event | 21 (11.3) | 18 (11.1) | 0.958 | 13 (8.6) | 16 (11.9( | 0.355 |

Supplementary table 4: Comparing the clinical characteristics and natural history of patients with and without a disease-causing variant in sarcomeric or non-sarcomeric gene identified on genetic testing

HCM = hypertrophic cardiomyopathy, SCD = sudden cardiac death, NYHA = New York heart association, NSVT = non-sustained ventricular tachycardia, LVMWT = left ventricular maximal wall thickness, SD = standard deviation, IQR = interquartile range, LA = left atrial, LVOT = left ventricular outflow tract, SCD = sudden cardiac death

|  | **Pre-adolescent** | | | **Adolescent** | | |
| --- | --- | --- | --- | --- | --- | --- |
|  | **Disease causing sarcomeric variant (P/LP) (n=175)** | **No disease causing sarcomeric variant**  **(n=162)** | **P value** | **Disease causing sarcomeric variant (P/LP) (n=148)** | **No disease causing sarcomeric variant**  **(n=134)** | **P value** |
| Age at baseline | 6.5 (3.4) | 6.4 (3.4) | 0.8722 | 13.9 (1.4) | 13.7 (1.2) | 0.304 |
| Male sex | 108 (61.7) | 112 (69.1) | 0.153 | 99 (66.9) | 99 (73.9) | 0.200 |
| FHx HCM | 125 (72.3) | 72 (45.0) | <0.001 | 104 (71.2) | 56 (42.1) | <0.001 |
| FHx SCD | 20 (11.4) | 17 (10.5) | 0.784 | 31 (21.0) | 17 (12.6) | 0.061 |
| Unexplained syncope | 10 (5.7) | 9 (5.6) | 0.950 | 18 (12.2) | 18 (13.3) | 0.768 |
| NYHA/Ross>1 | 27 (15.6) | 34 (21.4) | 0.405 | 29 (20.3) | 33 (25.0) | 0.551 |
| NSVT | 6 (4.2) | 7 (5.1) | 0.866 | 6 (4.3) | 7 (5.6) | 0.713 |
| B Blockers | 56 (32.2) | 64 (39.5) | 0.209 | 59 (39.9) | 63 (46.7) | 0.277 |
| Z score MWT | 10.8 (7.7) | 9.9 (6.7) | 0.8567 | 12.5 (7.0) | 10.7 (6.6) | 0.986 |
| Z score LA | -0.5 (4.3) | -1.3 (4.9) | 0.940 | -0.1 (4.0) | -0.7 (4.4) | 0.880 |
| LVOT gradient | 9 (6,20) | 10 (5, 35)) | 0.1407 | 8 (5,15) | 10 (5, 21) | 0.2362 |
| LVOTO | 31 (21.5) | 42 (30.4) | 0.088 | 23 (16.8) | 25 (21.0) | 0.388 |
| Death | 4 (2.3) | 5 (3.1) | 0.649 | 3 (2.0) | 6 (4.4) | 0.247 |
| Life threatening arrhythmic event | 21 (12.0) | 18 (11.1) | 0.799 | 12 (8.1) | 16 (11.9) | 0.292 |

Supplementary table 5: Comparing the clinical characteristics and natural history of patients with and without a disease-causing sarcomeric variant in identified on genetic testing

HCM = hypertrophic cardiomyopathy, SCD = sudden cardiac death, NYHA = New York heart association, NSVT = non-sustained ventricular tachycardia, LVMWT = left ventricular maximal wall thickness, SD = standard deviation, IQR = interquartile range, LA = left atrial, LVOT = left ventricular outflow tract, SCD = sudden cardiac death

|  | Univariable Cox regression analysis | | | Multivariable Cox regression analysis |  |  |
| --- | --- | --- | --- | --- | --- | --- |
|  | Hazard ratio | 95% CI | P value | Hazard ratio | 95% CI | P value |
| Increasing age | 1.09 | 1.01 – 1.18 | 0.022 |  |  |  |
| FHx HCM | 1.04 | 0.63 – 1.72 | 0.865 |  |  |  |
| NYHA >1 | 4.28 | 2.60 – 7.06 | <0.001 | 2.20 | 0.90 – 5.44 | 0.085 |
| LVOT gradient | 1.002 | 0.99 – 1.01 | 0.589 |  |  |  |
| LA z score | 1.15 | 1.09 – 1.22 | <0.001 | 1.21 | 1.07 – 1.36 | 0.020 |
| LVMWT z score | 1.003 | 0.97 – 1.04 | 0.859 |  |  |  |
| Disease causing variant | 0.40 | 0.16 – 0.99 | 0.048 | 0.37 | 0.15 – 0.90 | 0.029 |
| B-blocker therapy | 1.16 | 0.70 – 1.93 | 0.562 |  |  |  |

Supplementary table 6: Cox regression analysis of baseline clinical features associated with mortality of cardiac transplant

Supplementary figure 1: Participation recruitment and retention in the International Paediatric Hypertrophic Cardiomyopathy Consortium

Supplementary figure 2: Flow sheet showing genetic testing strategy

*Nucleic acid or amino acid change not provided by participating centres
